# Ni–citric acid coordination polymer as a practical catalyst for multicomponent reactions

**DOI:** 10.1038/s41598-021-03857-w

**Published:** 2021-12-28

**Authors:** Mostafa Koolivand, Mohsen Nikoorazm, Arash Ghorbani-Choghamarani, Reza Azadbakht, Bahman Tahmasbi

**Affiliations:** 1 Department of Chemistry, Faculty of Science, Ilam University, P. O. Box 69315516, Ilam, Iran; 2 Department of Organic Chemistry, Faculty of Chemistry, Bu-Ali Sina University, 6517838683 Hamedan, Iran

**Keywords:** Catalysis, Catalyst synthesis, Heterogeneous catalysis

## Abstract

Coordinative polymers (CP) are a subclass of Metal–organic frameworks (MOFs) with porous microstructures which have been widely synthesized in recent years and applied in various fields especially in catalysis science. In this work Coordinative polymers (CP) of nickel and citric acid (CA) was prepared as a new catalyst (Ni-CP) and applied in organic multicomponent reactions. The obtained catalyst was characterized by SEM, WDX, EDS, AAS, FT-IR, XRD and BET analysis. N_2_ adsorption–desorption isotherms indicate good BET surface area for Ni-CP; therefore can be employed as an efficient catalyst in multicomponent reactions for the synthesis of polyhydroquinoline and 2,3-dihydroquinazolin-4(1H)-one derivatives. Finally, this catalyst was recovered and reused several consecutive times.

## Introduction

Multicomponent reactions (MCRs) have gained more attention in the last decade as a great and powerful strategy in the synthesis of natural products, medicinal chemistry and organic reactions^1–11^. Typically, MCRs are defined as one-pot reactions with more than two starting materials join to generate a desired single product of the reagent atoms^1,12^. Comparison to multistep reactions, MCRs offer a high atom economy, simple procedures, selectivity, environmental friendliness, time and energy saving^8,9,13^. In this regard various heterogeneous and homogeneous (Transition metals, transition metal complexes or supported metals) catalysts are employed in MCRs to increase selectivity and rate of the reaction^4,14^. For example, multicomponent reactions have been significantly extended using Coordinative polymers (CP)^15^. Coordinative polymers are commonly formed by transition metal ions and an organic multi-dentate ligand^15,16^. CP platforms with unique properties such as easy recoverability, large pore aperture, low density, high specific surface area, permanent nanoscale porosity and uniform structured cavities, have been used as ideal catalysts in MCRs and other applications such as drug delivery, gas storage, separation science, gas purification, sensing, optoelectronics, magnetism and luminescence^17–22^. In the continuation of the development of new CP catalysis in MCRs. Herein we report the synthesis of a new CP of nickel and citric acid and its catalytic activity in the synthesis of polyhydroquinoline and 2,3-dihydroquinazolin-4(1H)-one derivatives. Polyhydroquinolines and 2,3-dihydroquinazolin-4(1H)-ones have a wide range of biological properties and pharmaceutical activities^23–28^. For example, nifedipine, amlodipine and nicardipine are several biologically active compounds of polyhydroquinoline derivatives^29,30^. Besides, 2, 3-Dihydroquinazolin-4(1H) ones are known to possess diverse pharmacological actions^31,32^. For example, quinethazone, fenquizone, metolazone, evodiamine, afloqualone, methaqualone are several biologically active compounds of 2,3-dihydroquinazolin-4(1H)-one derivatives^33–35^. Also, 2-(2-hydroxy-phenyl)-4(3H)-quinazolinone (HPQ) was utilized in the detection of metal ions or act as a biosensor to scrutinize the Monoamine oxidases activity^36–39^.

## Experimental 

### Materials and instruments

All chemicals and solvents employed in this work were purchased from Aldrich or Merck companies and used without further purification.

### Preparation of Ni-CP

**Figure 1:**
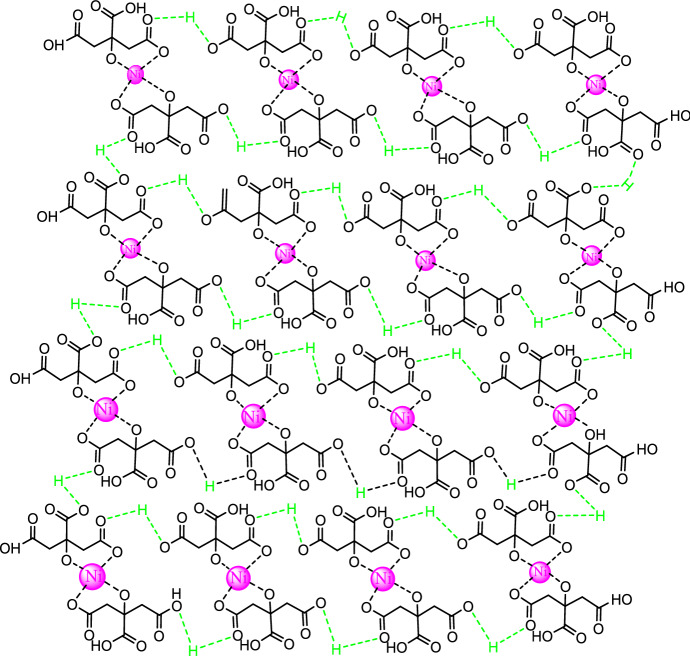
The topological structure of Ni-CP.

To prepare Ni-CP, citric acid (1 mmol) was dissolved in water (2 mL) and, then, it was added to a solution of DMF (12 mL) containing 2 mmol of nickel nitrate. Afterward, the obtained mixture was transferred into a autoclave and heated at 160 °C for 1 day, which then cooled down and Subsequently washed with ethyl acetate. Finally, the Ni-CP product was dried at 60 °C in an oven (Fig. 1).

### General procedure for the synthesis of polyhydroquinoline in the presence of Ni-CP

A mixture of various aromatic aldehydes (1 mmol), NH_4_OAc (1 mmol), dimedone (1 mmol), ethyl acetoacetate (1 mmol), and Ni-CP (5 mg), was refluxed in ethanol at 80 °C. Completion of the following reaction has been analyzed via TLC, Subsequent the reaction mixture was diluted with hot ethanol to dissolve the organic products, the catalyst was separated using simple filtration and the resultant unrefined solid products, was further purified through recrystallization in ethanol and dried to obtain a pure solid.

### General procedure for the synthesis of 2,3-dihydroquinazolin-4(1H)-ones in the presence of Ni-CP

A mixture of various aromatic aldehydes (1 mmol), anthranilamide (1 mmol) and Ni-CP (6 mg), was refluxed in ethanol at 80 °C. Completion of the following reaction has been analyzed via TLC, Subsequent the reaction mixture was diluted with hot ethanol to dissolve the organic products, the catalyst was separated using simple filtration and the resultant unrefined solid products, was further purified through recrystallization in ethanol and dried to obtain a pure solid.

### Selected spectral data

#### 2-(4-Methoxyphenyl)-2,3-dihydroquinazolin-4(1H)-one (Table 5, entry 4)

^1^H NMR (400 MHz, CDCl_3_): δ_H_ = 8.18 (s, 1H), 7.62–7.60 (d, *J* = 8 Hz, 1H), 7.42–7.41 (d, *J* = 4 Hz, 2H), 7.25–7.22 (t, *J* = 8 Hz, 1H), 7.00 (br, 1H), 6.95–6.93 (d, *J* = 8 Hz, 2H), 6.75–6.73 (d, *J* = 8 Hz, 1H), 6.68–6.65 (t, *J* = 8 Hz, 1H), 5.70 (s, 1H), 3.74 (s, 3H) ppm.

#### 2-(p-tolyl)-2,3-Dihydroquinazolin-4(1H)-one (Table 5, entry 3)

^1^H NMR (400 MHz, CDCl_3_): δ_H_ = 8.23 (s, 1H), 7.63–7.61 (d, *J* = 8 Hz, 1H), 7.39–7.37 (d, *J* = 4 Hz, 2H), 7.25–7.22 (t, *J* = 4 Hz, 1H), 7.19–7.18 (d, *J* = 4 Hz, 1H), 7.05 (s, 1H), 6.76–6.74 (d, *J* = 8 Hz, 1H), 6.69–6.65 (t, *J* = 8 Hz, 1H), 5.72 (s, 1H), 2.29 (s, 3H) ppm.

#### 2-Phenyl-2,3-Dihydroquinazolin-4(1H)-one (Table 5, entry 1)

^1^H NMR (400 MHz, CDCl_3_): δ_H_ = 8.27 (s, 1H), 7.62–7.60 (d, *J* = 8 Hz, 1H), 7.50–7.48 (d, *J* = 4 Hz, 2H), 7.40–7.34 (m, 3H), 7.25–7.22 (t, *J* = 8 Hz, 1H), 7.10 (s, 1H), 6.75–6.74 (d, *J* = 4 Hz, 1H), 6.68–6.65 (t, *J* = 8 Hz, 1H), 5.75 (s, 1H) ppm.

#### Ethyl 2,7,7-trimethyl-4-(3-nitrophenyl)-5-oxo-1,4,5,6,7,8-hexahydroquinoline-3-carboxylate (Table 3, entry 8)

^1^H NMR (400 MHz, CDCl_3_): δ_H_ = 9.24 (s, 1H), 7.97 (s, 2H), 7.61–7.51 (d, *J* = 40 Hz, 2H), 4.96 (s, 1H), 3.96 (s, 2H), 2.50–2.43 (m, 2H), 2.33 (s, 3H), 2.20–2.17 (d, *J* = 12 Hz, 1H), 1.99–1.96 (d, *J* = 12 Hz, 1H), 1.12–1.09 (t, *J* = 8 Hz, 3H), 1.00 (s, 3H), 0.82 (s, 3H) ppm.

#### Ethyl 4-(4-methoxyphenyl)-2,7,7-trimethyl-5-oxo-1,4,5,6,7,8-hexahydroquinoline-3-carboxylate (Table 3, entry 3)

^1^H NMR (400 MHz, CDCl_3_): δ_H_ = 9.00 (s, 1H), 7.05–7.03 (d, *J* = 8 Hz, 2H), 6.74–6.72 (d, *J* = 8 Hz, 2H), 4.78 (s, 1H), 3.97–3.95 (d, *J* = 8 Hz, 2H), 3.66 (s, 3H), 2.49 (s, 2H), 2.26 (s, 3H), 2.16–2.13 (d, *J* = 12 Hz, 1H), 1.98–1.94 (d, *J* = 16 Hz, 1H), 1.14–1.11 (t, *J* = 8 Hz, 3H), 1.00 (s, 3H), 0.88 (s, 3H) ppm.

## Result and discussion

**Figure 2:**
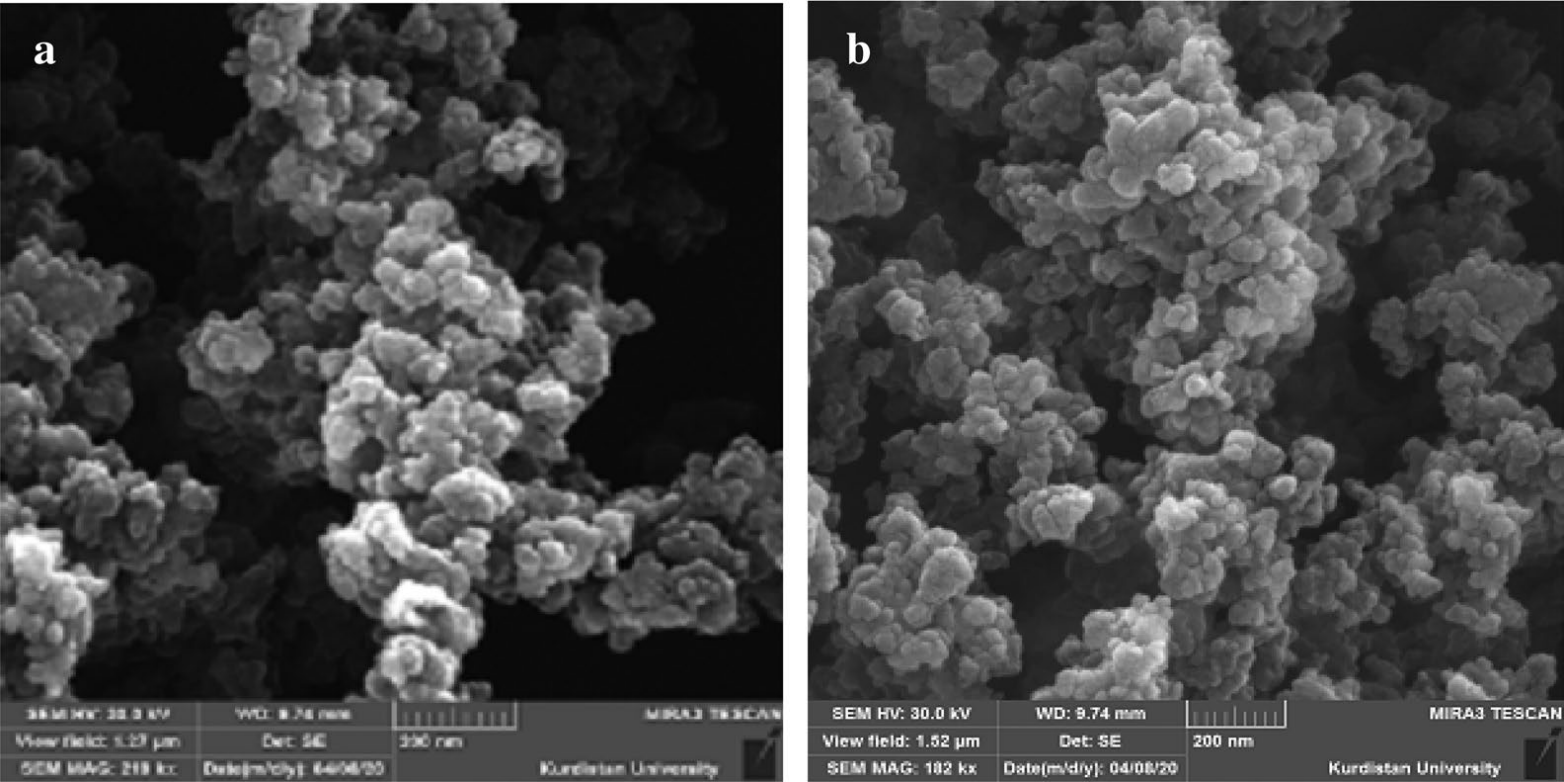
SEM images of Ni-CP.

Herein coordination polymer of nickel and Citric acid (Ni-CP) is reported as an efficient catalyst in multicomponent reactions for the synthesis of polyhydroquinoline and 2,3-dihydroquinazolin-4(1H)-one derivatives. Ni-CP was characterized by AAS, EDS, WDX, SEM, TGA, XRD, FT-IR and BET analysis. The surface morphological features of synthesized Ni-CP were studied by scanning electron microscope. Figure 2 shows the SEM image of Ni-CP with a magnification of 5000. The instrumental parameters, accelerating voltage, spot size, and magnification and working distances are indicated on the SEM image. The results indicate that mono-dispersive and highly crystalline Ni-CP is obtained. The appearance of some particles is spherical shape. We can observe that the Ni-CP is highly agglomerated and they are essentially cluster of Ni-CP. The SEM picture indicates the size of polycrystalline particles. The observation of some larger Ni-CP may be attributed to the fact that Ni-CP tends to agglomerate due to their high surface energy and high surface tension of the ultrafine Ni-CP. The fine particle size results in a large surface area that in turn, enhances the Ni-CP catalytic activity. So, we can conclude that the prepared Ni-CP particles are in the nanometer range. The average diameter of the particle observed from SEM analysis is 30 nm, which is larger than the diameter predicted from X-Ray broadening.

**Figure 3:**
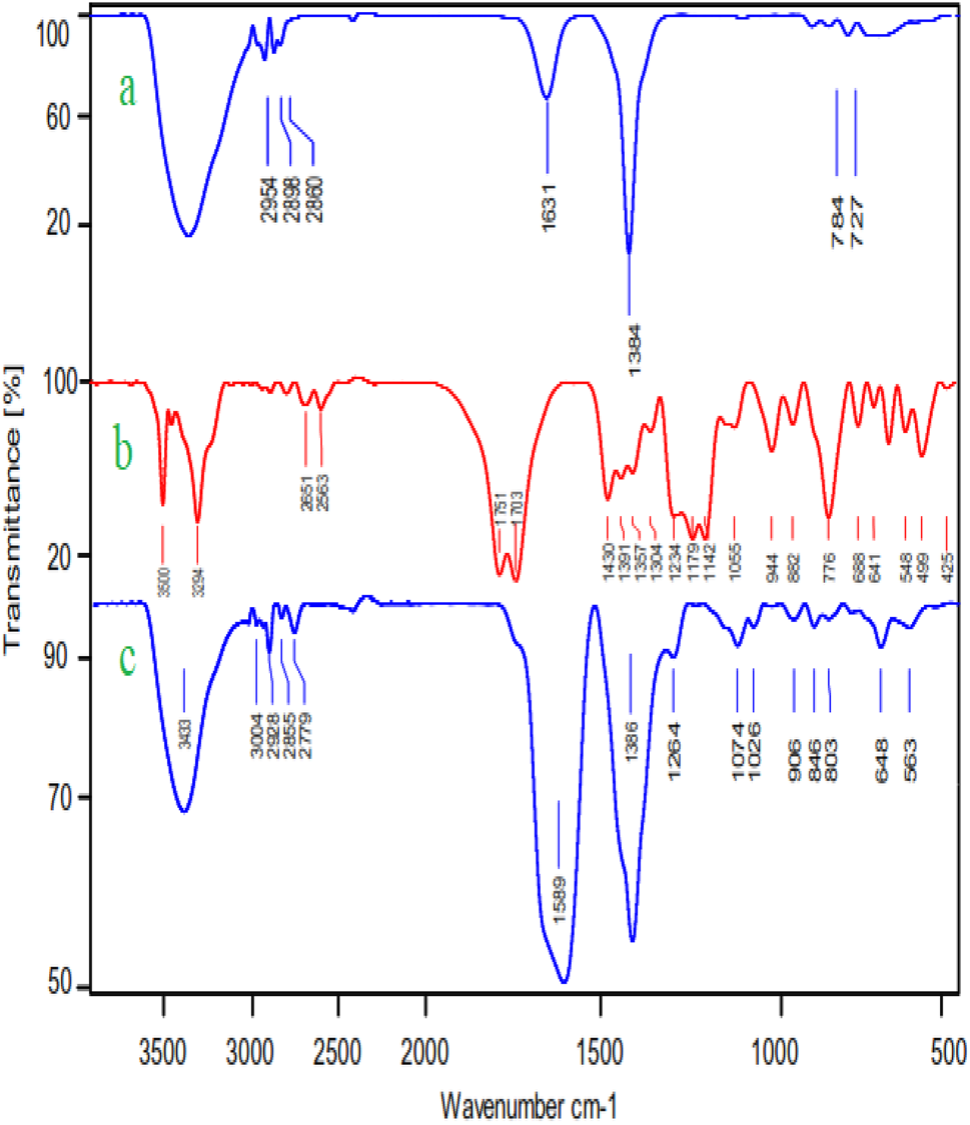
FT-IR Spectrums of (a) Nickel nitrate, (b) citric acid and (c) Ni-CP catalyst.

The FT-IR spectrum of the citric acid had absorption bands in the regions of 1703 cm^−1^ and 1751 cm^−1^ respectively, which is related to the stretching vibration of the C=O bonds of aliphatic carboxylic acid groups (Fig. 3b). These absorption peaks were not observed in the spectra of the prepared Ni-CP, while the spectra of the Ni-CP presented two absorption peaks related to the vibration of the C=O bonds of aliphatic carboxylic acid groups at 1589 cm^−1^. The high shift to the lower wavenumber of the absorption peak of carbonyl demonstrates the existence of the metal coordination bonding and also confirms the complexation reaction between Ni and citric acid (Fig. 3c). In addition, the peaks at the region of 2855–2928 cm^−1^ can be regarded as the characteristic of the stretching vibrations of aliphatic C–H^15^. The presence of aliphatic C–H stretching vibration indicates that the organic ligand used in the final sorbent structure remained and was not destroyed. In addition, various peaks were presented such as 400–1000 cm^−1^ for Ni–O bonds and 3000–3600 cm^−1^ for OH of carboxylic acid^40,41^. Based on the FT-IR results, we can also observe that the Ni-CP obtained from Nickel Nitrate shows sharp characteristic peaks, suggesting the high crystalline nature of Ni-CP (Fig. 3).

**Figure 4:**
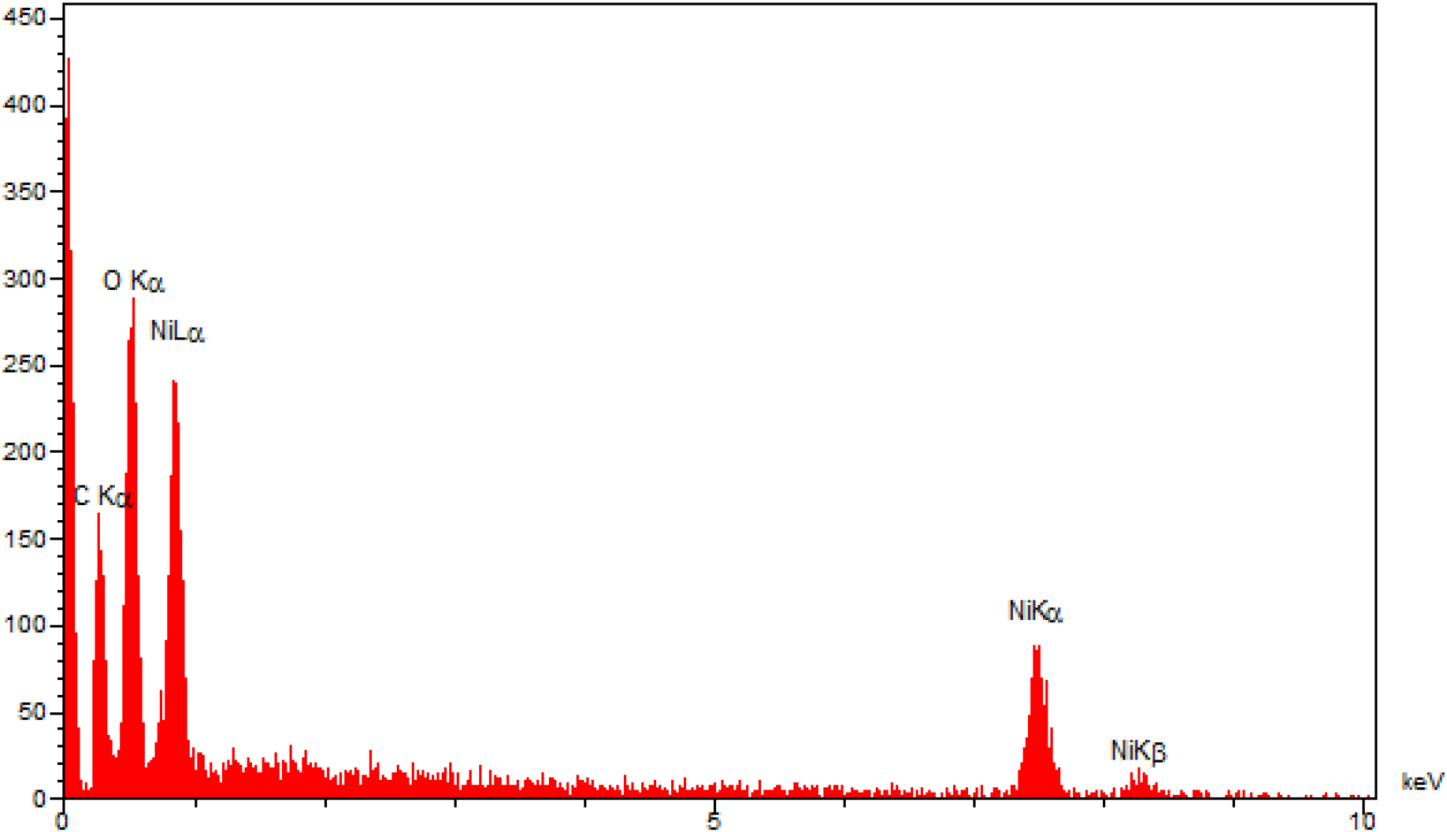
EDS diagram of Ni-CP.

The elemental content of Ni-CP was obtained by EDS (energy-dispersive X-ray spectroscopy) analysis (Fig. 4). Based on the EDS results, the presence of oxygen (A%:45), carbon (A%:47), and as well as nickel (A%:8) species have been confirmed in the structure of the catalyst.

**Figure 5:**
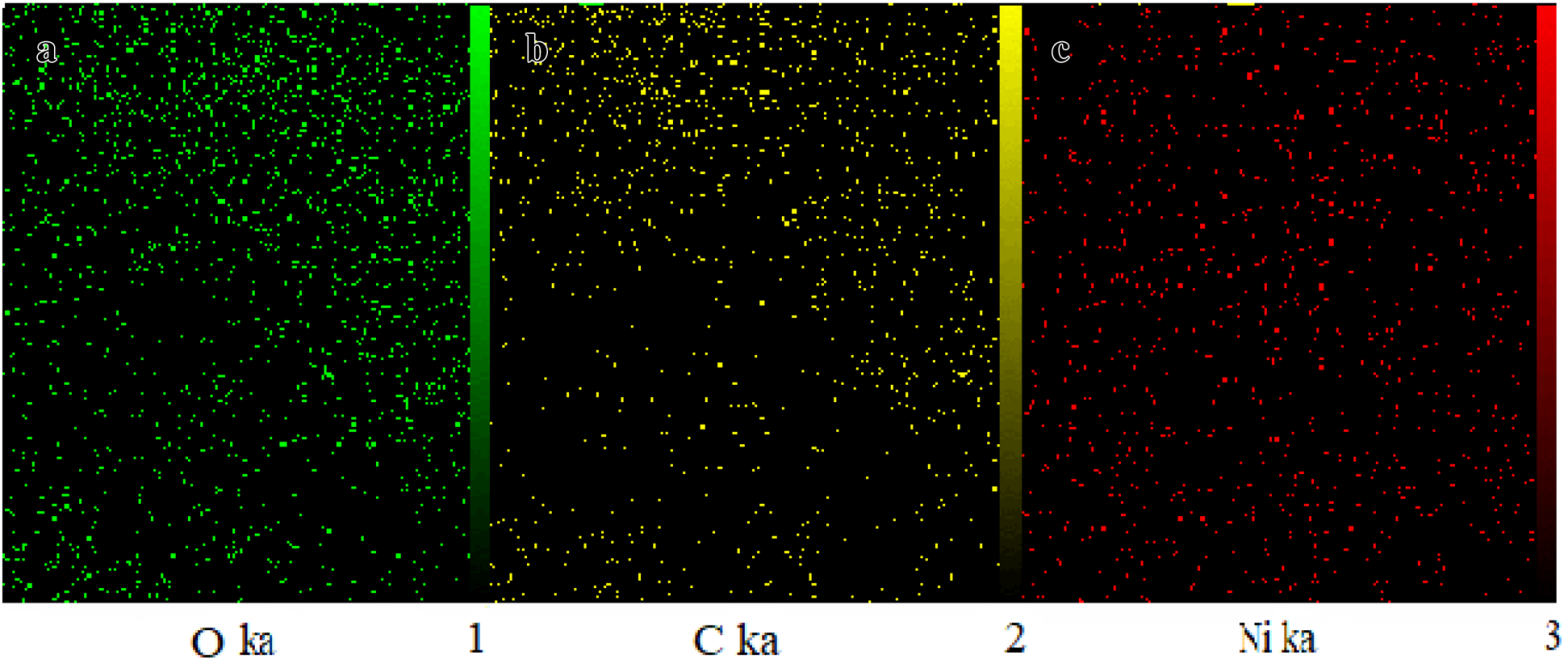
Elemental mapping of (**a**) oxygen, (**b**) carbon, and (**c**) nickel for Ni-CP.

The homogeneous distribution of these elements has been studied by X-Ray Mapping (WDX analysis) in this catalyst which is shown in (Fig. 5). Also, the exact amount of nickel in the structure of this CP catalyst was calculated by atomic absorption spectroscopy (AAS) analysis which was found to be 2.07 × 10^−3^ mol g^−1^.

**Figure 6:**
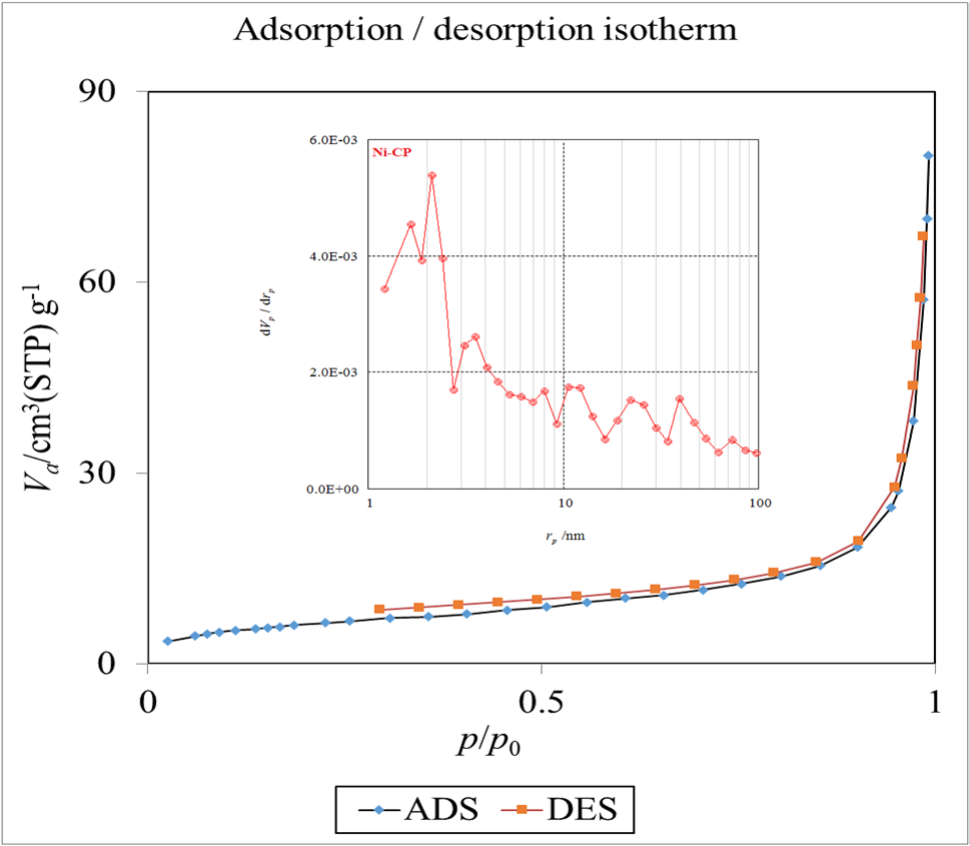
N_2_ adsorption–desorption isotherms of Ni-CP.

To investigate the porous nature and the efficient surface area of Ni-CP, nitrogen adsorption–desorption measurement (BET) was performed. The BET isotherms of Ni-CP and the corresponding Barrett–Joyner–Halenda (BJH) pore size distributions plot are shown in (Fig. 6). As could be seen from this Figure, the observed type-H3 hysteresis loop in the partial pressure range from 0.3 to 1.0, indicates the slit-shaped pores. The isotherm revealing type-H3 does not show any limiting adsorption at high P/P0, which is observed with spherical in shape particles^42^. Based on BET results, this CP catalyst has a high surface area of about 22.65 m^2^ g^−1^. Also, pore volumes and pore diameters of this CP catalyst are 0.11 cm^3^ g^−1^ and 19.34 nm respectively. The textural properties of Ni-CP which were obtained from N_2_ adsorption–desorption analysis are summarized in (Table 1).

**Table 1:** Textural properties of Ni-CP.

S_BET_ (m^2^ g^−1^)	Pore diameter (nm)	Pore volume (cm^3^ g^−1^)
22.65	19.34	0.11

**Figure 7:**
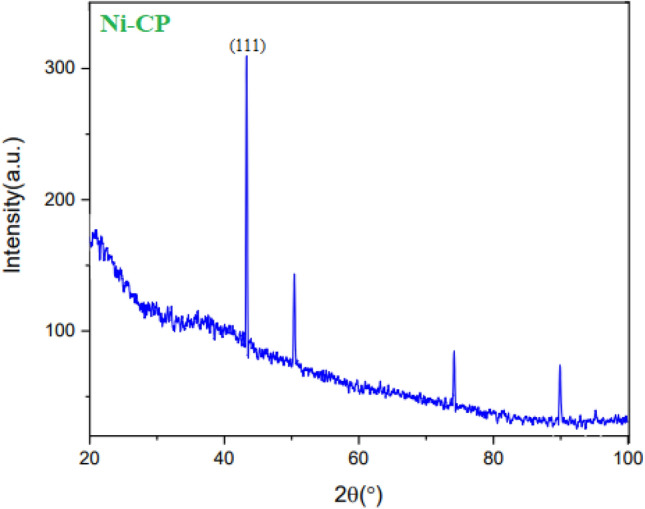
Normal XRD pattern of Ni-CP.

The synthesized CP materials were characterized by PXRD analysis using PW1730 instrument from Philips Company having CuKα (λ = 1.540598 Å) radiation at 40 kV and 30 mA with 2θ = 20°–100°. The XRD pattern of Ni-CP is shown in (Fig. 7). According to powder PXRD standards (PXRD, Ref. No. 01-087-0718), the crystalline peaks appearing at 42.2°, 51.3°, 75 °, 90.3° can be attributed to the (111), (300), (320), and (350) crystallographic planes of nickel crystals, which are in agreement with the previously reported works of literature ^43,44^. The PXRD patterns shown in (Fig. 7) confirm the successful coordination of nickel ions within the prepared framework. In addition, the (111) Ni diffraction peak with appreciable intensity further confirms the presence of Ni metal in the prepared Ni–CP^45^.

**Figure 8:**
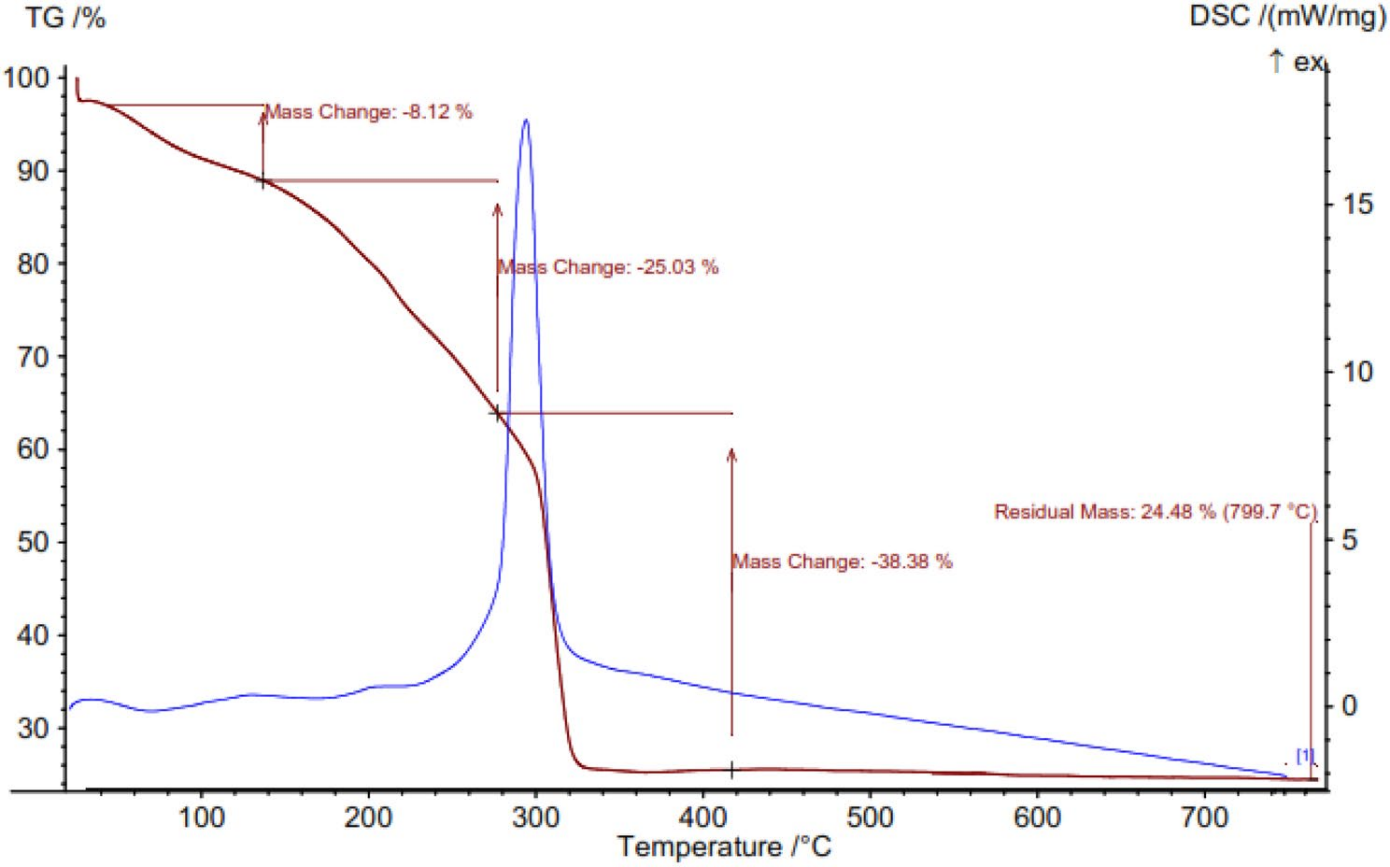
TGA diagram of Ni-CP.

The mass ratios and the thermal stability of Ni-CP were examined by the thermogravimetric analysis (TGA) (Fig. 8). In the TGA curve, the two obvious weight losses were found in the temperature range of 50–270 °C, which can be attributed to the release of the physically adsorbed moisture (water) and DMF solvents from the sample^15^. In this sense, it was at above 270 °C that the framework degradation started. The main weight loss at 270–340 °C was caused by the decomposition of citric acid ligand^46^. This result confirms the successful synthesis of Ni-CP and also indicates that the thermal stability of the sample is about 270 °C. The DSC results also support the TGA data, based on weight loss of the sample, and approve the range of temperature stability of sample.

### Catalytic study of Ni-CP

The catalytic activity of Ni-CP was investigated in the multicomponent reaction for the synthesis of polyhydroquinoline and 2,3-dihydroquinazolin-4(1H)-one derivatives. In the synthesis of polyhydroquinolines, 4-chlorobenzaldehyd was selected as a model substrate to obtain the best reaction conditions. The selected model reaction was tested in various conditions (Table 2). As shown, the model reaction did not proceed in the absence of Ni-CP (Table 2, entry 1). Meanwhile, the model reaction was completed in the presence of 5 mg of this catalyst with a 97% yield (Table 2, entry 2). Also, the model reaction was performed in a wide range of solvents and temperature. As shown, the reaction is dependent on solvent and temperature in which ethanol at 80 °C shows the best results for the synthesis of polyhydroquinolines (Table 2).

**Table 2:** Optimizing reaction conditions for the synthesis of polyhydroquinolines in the presence of Ni-CP.


Entry	Catalyst (mg)	Solvent	Temperature (°C)	Time (min)	Yield^a^ (%)
1	–	EtOH	Reflux	90	N.R^b^
2	5	EtOH	Reflux	90	97
3	5	EtOH: H_2_O (1:1)	80	90	86
4	5	Solvent free	80	90	90
5	5	PEG-400	80	30	78
6	5	DMSO	Reflux	90	84
7	2	EtOH	Reflux	90	67
8	4	EtOH	Reflux	30	89
9	6	EtOH	Reflux	30	97
10	5	EtOH	25	30	Trace
11	5	EtOH	50	30	48
12	5	EtOH	65	30	68
13	5	EtOH	70	30	84

^a^Isolated yield.

^b^No reaction.

To extend the application of Ni-CP as a catalyst, the various aldehydes were investigated under obtained optimizing reaction conditions (Table 3). All products having electron-withdrawing groups or electron-donating groups were synthesized with good to excellent yields. In this investigation, electron-donating groups (such as OH, Me, OMe and Nme_2_) and electron-withdrawing groups (such as Cl, F, Br, CN, NO_2_ and CF_3_) on the aromatic ring of benzaldehydes were examined under obtained optimizing reaction conditions for the synthesis of the wide range of polyhydroquinoline derivatives.

**Table 3:** Synthesis of polyhydroquinoline derivatives catalysed by Ni-CP.


Entry	aldehyde	product	Time (min)	Yield (%)^a,b^	M.P
					Measured
			90	97	240–243
			120	92	252–255
			110	95	251–253
			140	92	215–217
			95	95	202–204
			150	89	230–233
			115	96	247–250
			80	92	173–175
			85	87	169–172
			165	91	246–249

^a^Isolated yield.

^b^Reaction conditions: Aromatic aldehyde (1 mmol), dimedone (1 mmol), ethyl acetoacetate (1 mmol), ammonium acetate (1.2 mmol), Ni-CP (5 mg) in ethanol under reflux conditions.

In continuation, the catalytic activity of Ni-CP was examined for the synthesis of 2,3-dihydroquinazolin-4(1*H*)-one derivatives as an efficient, recyclable, stable, and commercially available CP catalyst. The synthesis of 2,3-dihydroquinazolin-4(1*H*)-ones via cyclocondensation of anthranilamide and aldehydes in the presence of Ni-CP is shown in Table 4. In order to find the best reaction conditions, cyclocondensation of 4-chlorobenzaldehyde and anthranilamide was selected as model reaction (Table 4). At first, the effect of solvent was studied in the model reaction, in which the highest yields in lower reaction times were obtained in ethanol solvent (Table 4, entry 2). Meanwhile, other solvents such as PEG, H_2_O, and DMF afforded low to moderate yields (Table 4, entries 3–5). Then, the amount of Ni-CP and the effect of temperature were also examined. As shown in Table 4, this reaction requires 6 mg of Ni-CP as a catalyst in the best conditions. Also, the best results were obtained at 80 °C. Meanwhile, lower yields were observed at 25 °C, 50 °C and 70 °C (Table 4, entries 10–12).

**Table 4:** Optimizing reaction conditions for the synthesis of 2,3-dihydroquinazolin-4(1H)-ones in the presence of Ni-CP.


Entry	Catalyst (mg)	Solvent	Temperature (ºC)	Time (min)	Yield (%)^a^
1	–	EtOH	Reflux	120	N.R^b^
2	6	EtOH	Reflux	45	96
3	6	DMF	Reflux	45	Trace
4	6	PEG-400	Reflux	45	89
5	6	H_2_O	Reflux	45	66
6	1	EtOH	Reflux	45	Trace
7	3	EtOH	Reflux	45	58
8	5	EtOH	Reflux	45	88
9	8	EtOH	Reflux	45	96
10	6	EtOH	25	45	Trace
11	6	EtOH	50	45	51
12	6	EtOH	70	45	87

^a^Isolated yield.

^b^No reaction.

After optimization of the reaction conditions, we have investigated the synthesis of a series of 2,3-dihydroquinazolin-4(1H)-ones to explore the scope of this procedure (Table 5). In these studies, various functional groups on the aromatic ring of an aldehyde such as OH, Me, OMe, Cl, Br, F and NO_2_ were well tolerated. Therefore, this procedure is efficient for the synthesis of a wide range of 2,3-dihydroquinazolin-4(1H)-ones including electron-donating and electron- withdrawing substituents.

**Table 5:** Synthesis of 2,3-dihydroquinazolin-4(1H)-ones derivatives in the presence of Ni-CP.


Entry	Substrate	Product	Time (min)	Yield (%)^a^	Melting point
1			95	90	165–167
2			45	96	191–193
3			110	88	215–219
4			120	85	183–184
5			360	88	196–198
6			120	93	273–275
7			90	92	200–203
8			540	91	175–177
9			180	88	195–197

^a^Isolated yield.

^b^Reaction conditions: 4-Chlorobenzaldehyde (1 mmol), Anthranilamide (2-aminobenzamide) (1 mmol), Ni-CP (6 mg) in ethanol under reflux conditions.

**Figure 9:**
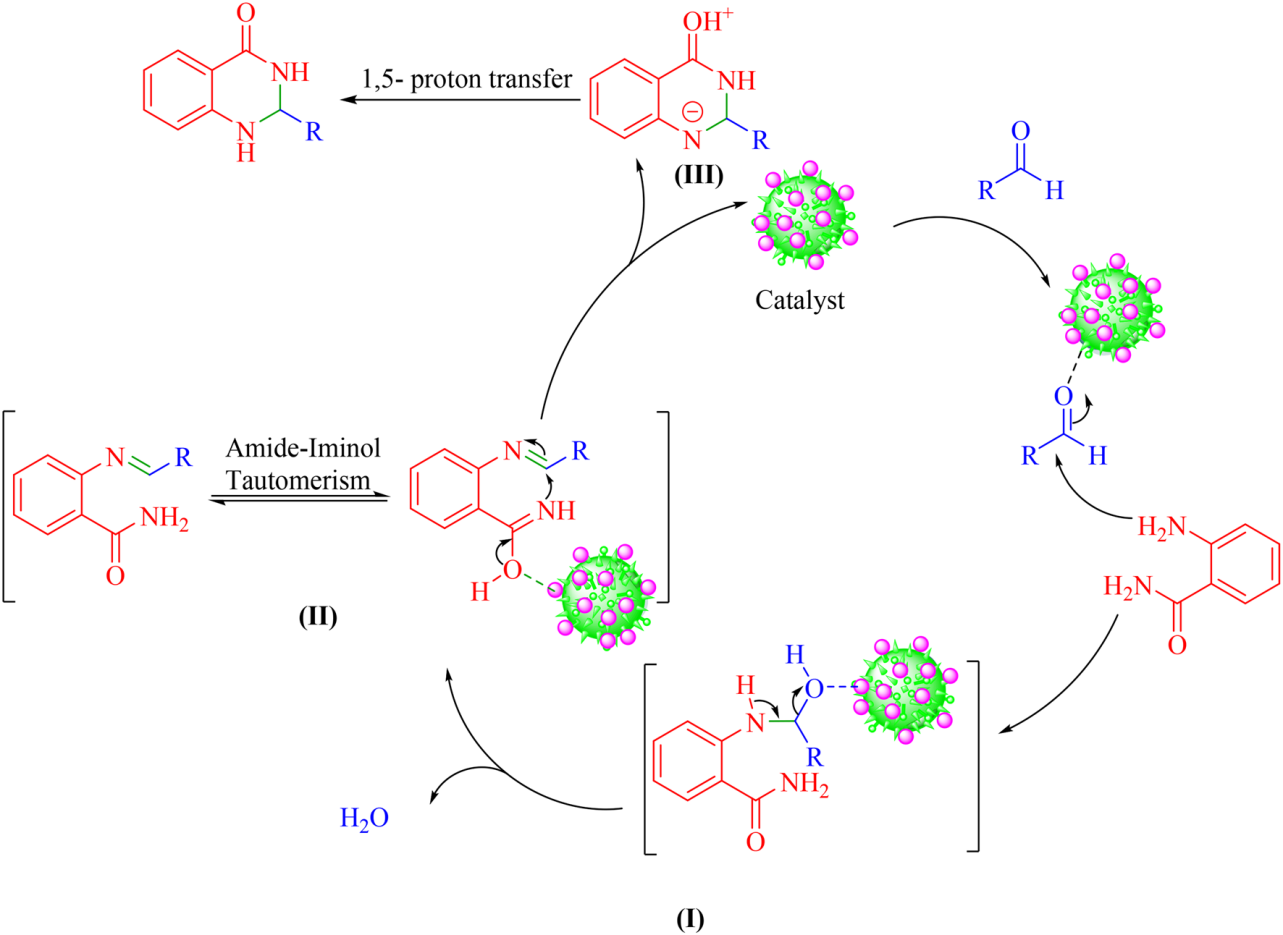
Proposed mechanism for the synthesis of 2,3-dihydroquinazolin-4(1H)-ones catalyzed by Ni-CP.

Based on the previous reports, the possible mechanism for the synthesis of 2,3-dihydroquinazolin-4(1H)-one derivatives in the presence of Lewis acid can be suggested in (Fig. 9)^28^. This mechanism has several steps in which the first step involves activation of aldehyde by Ni-CP catalyst. The second step includes attacking active aldehyde by the amine group of 2-amino benzamide which followed by the dehydration reaction led to the synthesis of imine **I**. Intermediate **I** is converted to intermediate **II** by an intermolecular reaction. This step is included tautomerization of the amide and an intramolecular nuclear attack on carbon of imine. Finally, 2,3-dihydroquinazolin-4(1H)-one derivatives were synthesized by the protons transfer and Ni-CP catalyst was regenerated for new cycle^28^.

### Recycling ability and leaching study of the catalyst

**Figure 10:**
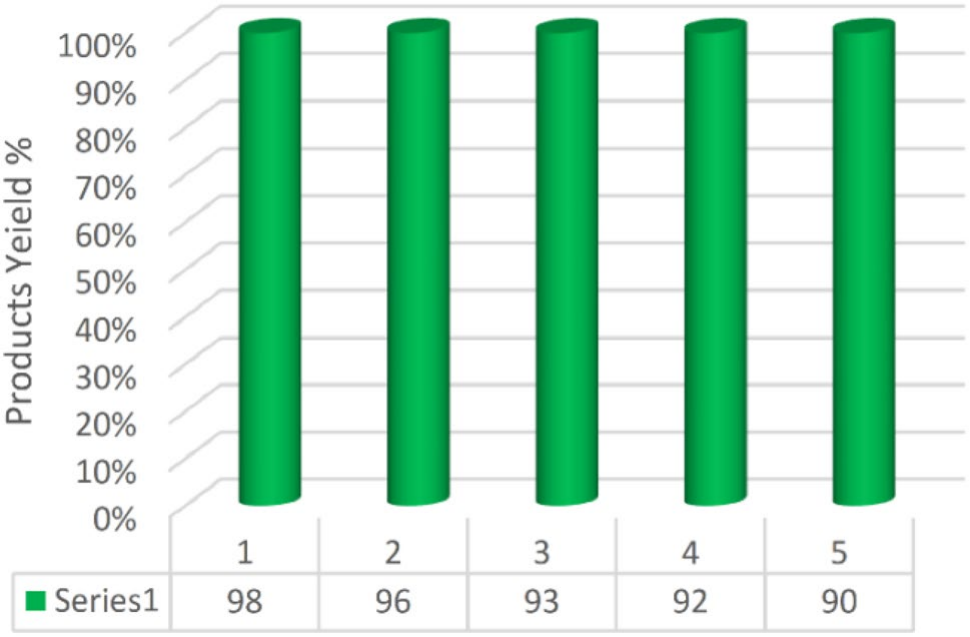
Recyclability of Ni-CP catalyst.

Recyclability is one of the most important properties of heterogeneous catalysts. The recyclability of the Ni-CP catalyst was analyzed in a typical [2+3] cycloaddition of anthranilamide and 4-chloro-benzaldehyde under the optimized reaction conditions. Figure 10 shows that Ni-CP catalyst can be reused up to 5 times without any significant loss of its activity.

**Figure 11:**
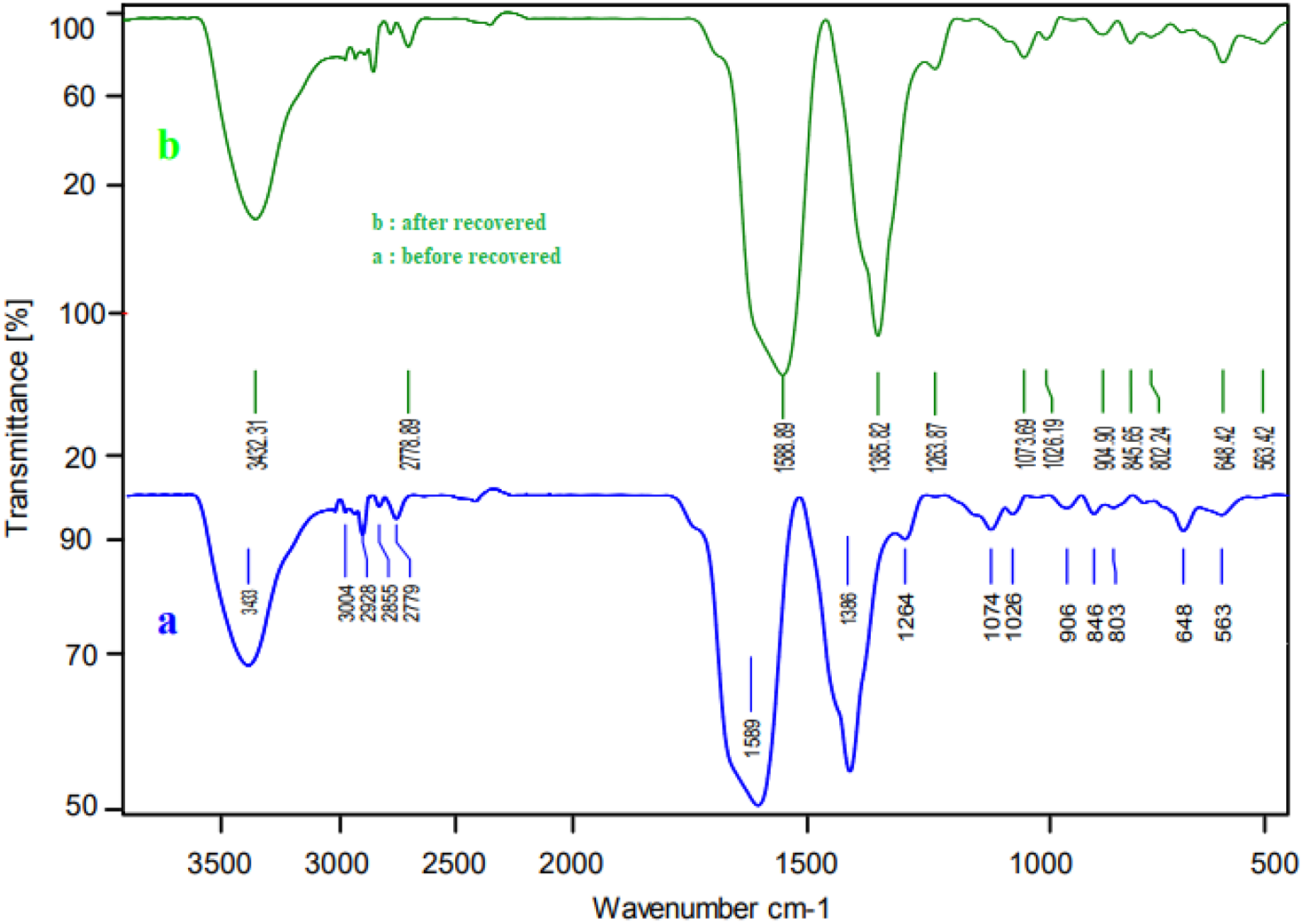
FT-IR analysis of Ni-CP catalyst.

**Figure 12:**
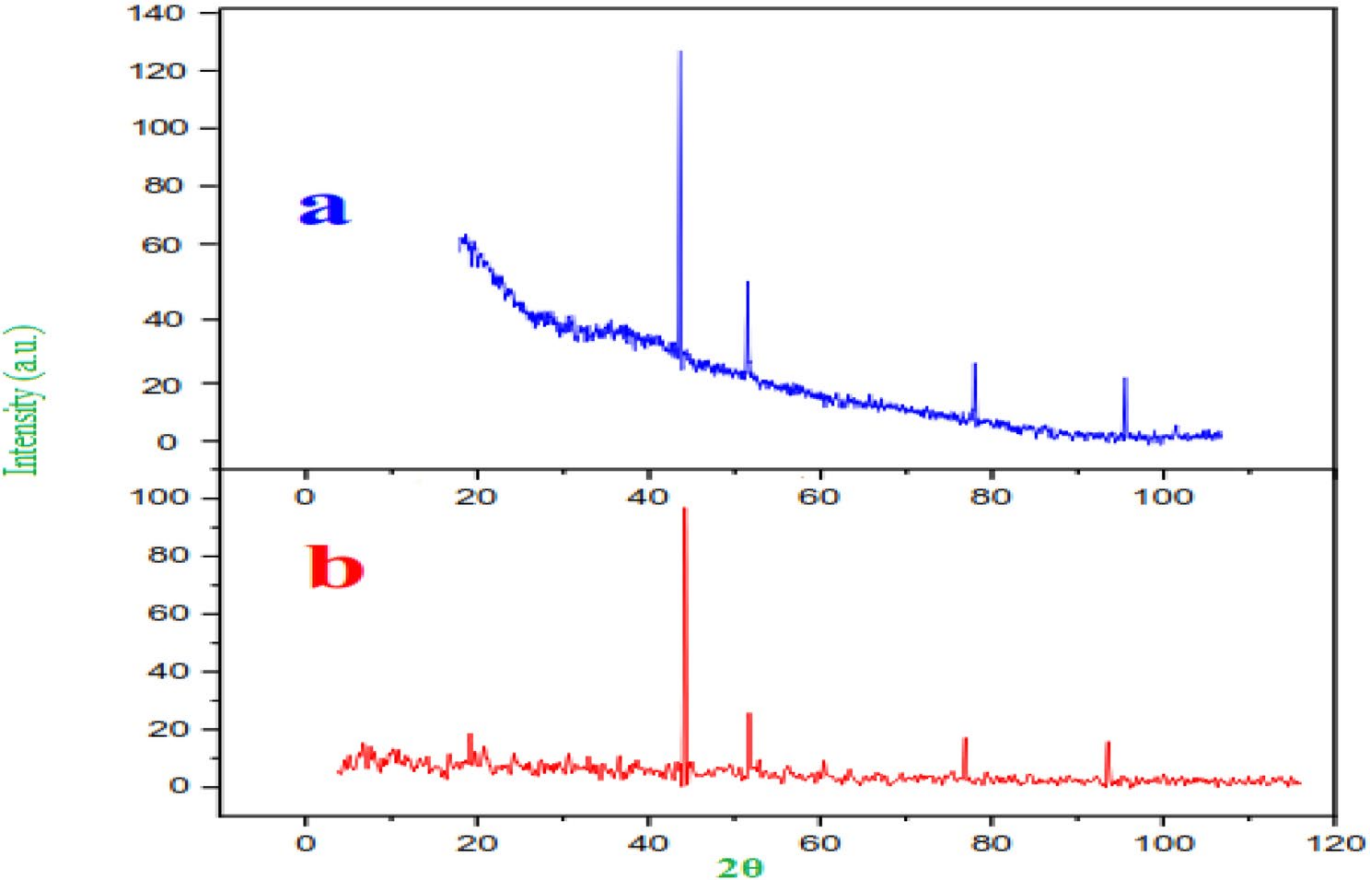
P-XRD analysis of Ni-CP catalyst.

The structure of reused catalyst was considered using FT-IR and XRD analysis. The FT-IR (Fig. 11) and XRD (Fig. 12) did not show any significant change compared to the fresh catalyst. All of the peaks of fresh catalyst are existed in the recovered catalyst.

In order to indicate the heterogeneous nature of Ni-CP, nickel leaching of this catalyst was studied. In this regard, the synthesis of 2,3-dihydroquinazolin-4(1*H*)-one using condensation of 4-chlorobenzaldehyde with 2-amino benzamide was selected as a model reaction. After completion of the reaction, the catalyst was removed by simple filtration and the amount of nickel was calculated in filtered solution by AAS which was found to be 0.0000370 mmol mL^−1^. As known, this very small amount of nickel doesn’t have a significant effect on the reaction progress. Therefore, the completion of the reaction could be dependent on the heterogeneous nickel species. It was also proved by the hot filtration test that the Ni-CP played a catalytic role in the reaction without the Ni leaching into the solution.

### Comparison of the catalyst

The efficiency of Ni-CP was investigated by comparison of our results on the synthesis of Polyhydroquinolines and 2,3-dihydroquinazolin-4(1H)-ones model reactions with the previous methods (Table 6). The products were obtained in higher yields over faster times in the presence of Ni-CP. Also, this catalyst has several advantages in terms of non-toxicity, price, stability and easy separation.

**Table 6:** Comparison results of Ni-CP with other catalysts in the synthesis of Polyhydroquinolines and 2,3-dihydroquinazolin-4(1H)-one.

Entry	Reaction	Catalyst	Time (min)	Yield (%)^a^	Ref.
1	Polyhydroquinoline	FeAl_2_O_4_	180	90	^26^
2	Polyhydroquinoline	CoFe_2_O_4_@Pr	145	96	^47^
3	Polyhydroquinoline	Fe_3_O_4_@D-NH-(CH_2_)_4_-SO_3_H	90	86	^48^
4	Polyhydroquinoline	AIL-SCMNPs	15	80	^49^
5	Polyhydroquinoline	Fe_3_O_4_@GA@IG	45	89	^50^
6	Polyhydroquinoline	Nickel nitrate	90	52	This work
7	Polyhydroquinoline	Ni-CP	90	97	This work
8	2,3-Dihydroquinazolin-4 (1H)-one	α-D-glucose	180	61	^51^
9	2,3-Dihydroquinazolin-4 (1H)-one	SBA-16/GPTMS-TSC-Cu^I^	35	95	^52^
10	2,3-Dihydroquinazolin-4 (1H)-one	CoFe_2_O_4_@Pr	60	97	^28^
11	2,3-Dihydroquinazolin-4 (1H)-one	Amberlyst-15	60	85	^53^
12	2,3-Dihydroquinazolin-4 (1H)-one	Nickel nitrate	45	63	This work
13	2,3-Dihydroquinazolin-4 (1H)-one	Ni-CP	45	96	This work

## Conclusion

In this work, nickel nitrate and citric acid nitrate were used to synthesis a new CP catalyst (Ni-CP). This CP catalyst was characterized by AAS, WDX, EDS, SEM, TGA, XRD and N_2_ adsorption–desorption analysis. Ni-CP was successfully described as an efficient and recyclable catalyst in the synthesis of polyhydroquinoline and 2,3-dihydroquinazolin-4(1H)-one derivatives in which all products were prepared in good yields. This catalyst has several advantages such as non-toxicity, price, stability and easy separation.

## Supplementary Information

Supplementary Information.
