# Supplementary material for: Ni–citric acid coordination polymer as a practical catalyst for multicomponent reactions
Source: Sci Rep. 2021 Dec 28;11:24475. doi: 10.1038/s41598-021-03857-w (PMC8714820; doi:10.1038/s41598-021-03857-w)
Supplement: Supplementary file 1 — Supplementary Information. [file 41598_2021_3857_MOESM1_ESM.doc]

Supplementary Data

**Ni-Citric acid coordination polymer as a practical catalyst for multicomponent reactions**

Mostafa Koolivand^a^, Mohsen Nikoorazm[[1]](#footnote-2)*,a, Arash Ghorbani-Choghamarani^*b^, Reza Azadbakht^b^, Bahman Tahmasbi^a^

^a^ Department of Chemistry, Faculty of Science, Ilam University, P. O. Box 69315516, Ilam, Iran.

^b^ Department of Organic Chemistry, Faculty of Chemistry, Bu-Ali Sina University, Hamedan 6517838683, Tel: +988138282807, Fax: +988138380709 Iran.

**Abstract:**

Coordinative polymers (CPs) are a subclass of Metal-organic frameworks (MOFs) with porous microstructures which have been widely synthesized in recent years and applied in various fields especially in catalysis science. In this work Coordinative polymers (CPs) of nickel and citric acid (CA) was prepared as a new catalyst (Ni-CPs) and applied in organic multicomponent reactions. The obtained catalyst was characterized by SEM, WDX, EDS, AAS, FT-IR, XRD and BET analysis. N_2_ adsorption-desorption isotherms indicate good BET surface area for Ni-CPs; therefore can be employed as an efficient catalyst in multicomponent reactions for the synthesis of polyhydroquinoline and 2,3-dihydroquinazolin-4(1H)-one derivatives. Finally, this catalyst was recovered and reused several consecutive times.

**Keywords:** Coordinative polymers, Citric acid, heterogeneous catalyst, polyhydroquinolines, 2, 3-dihydroquinazolin-4(1H)-ones.

**2-(4-methoxyphenyl)-2,3-dihydroquinazolin-4(1H)-one:** ^1^H NMR (400 MHz, CDCl_3_): δ_H_= 8.18 (s, 1H), 7.62-7.60 (d, *J*= 8 Hz, 1H), 7.42-7.41 (d, *J*= 4 Hz, 2H), 7.25-7.22 (t, *J*= 8Hz, 1H), 7.00 (br, 1H), 6.95-6.93 (d, *J*= 8 Hz, 2H), 6.75-6.73 (d, *J*= 8 Hz, 1H), 6.68-6.65 (t, *J*= 8Hz, 1H), 5.70 (s, 1H), 3.74 (s, 3H) ppm (Figure S1).


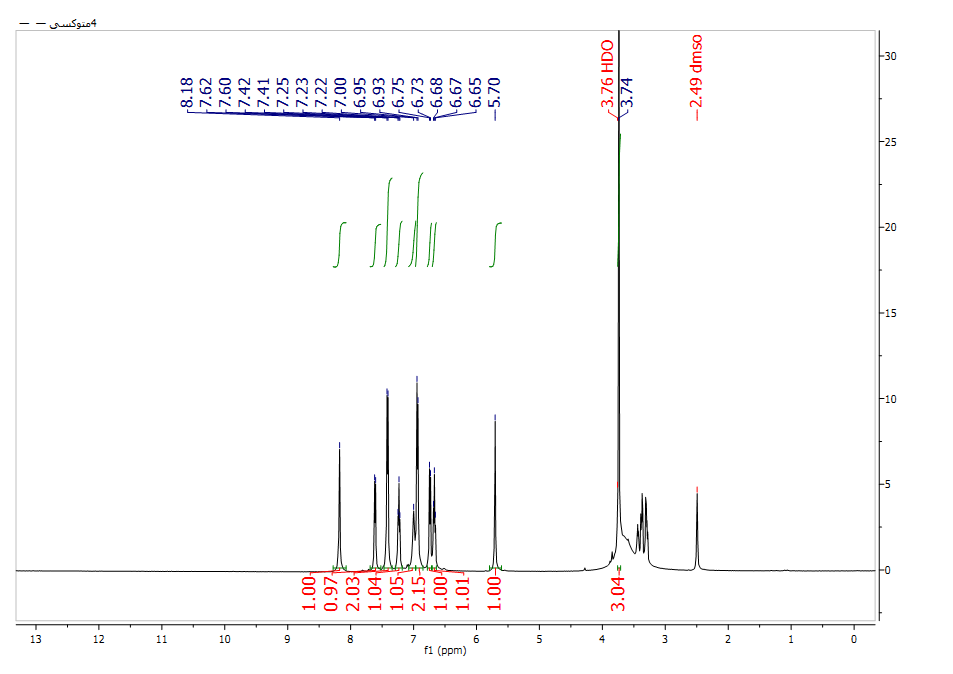


**Figure S1. ^1^H-NMR of 2-(4-methoxyphenyl)-2,3-dihydroquinazolin-4(1H)-one**

**2-(p-tolyl)-2,3-dihydroquinazolin-4(1H)-one:** ^1^H NMR (400 MHz, CDCl_3_): δ_H_= 8.23 (s, 1H), 7.63-7.61 (d, *J*= 8 Hz, 1H), 7.39-7.37 (d, *J*= 4 Hz, 2H), 7.25-7.22 (t, *J*= 4Hz, 1H), 7.19-7.18 (d, *J*= 4 Hz, 1H), 7.05 (s, 1H), 6.76-6.74 (d, *J*= 8 Hz, 1H), 6.69-6.65 (t, *J*= 8Hz, 1H), 5.72 (s, 1H), 2.29 (s, 3H) ppm (Figure S2).


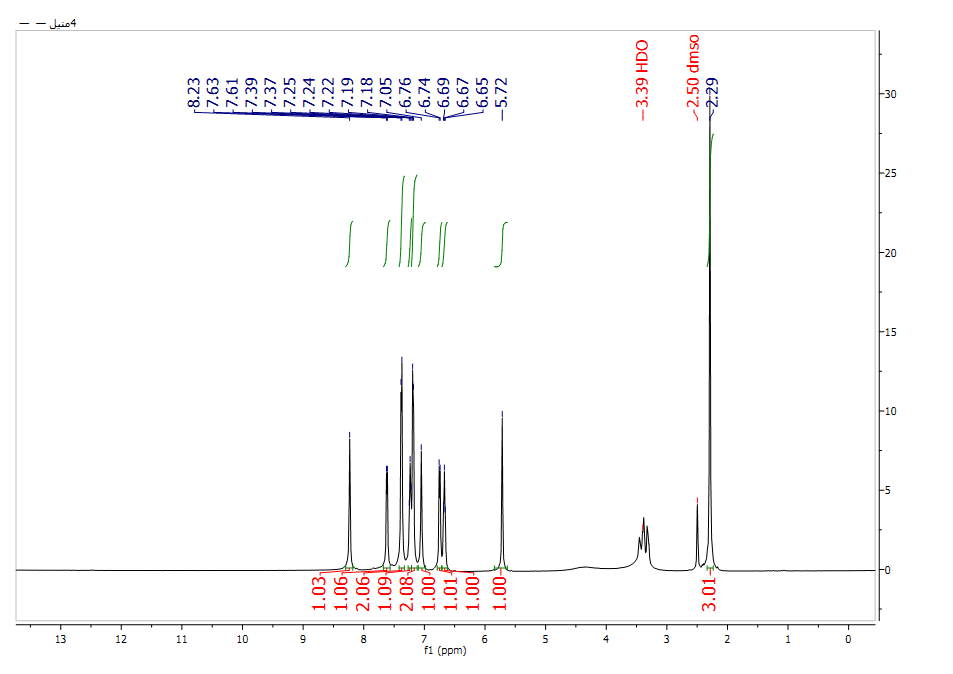


**Figure S2. ^1^H-NMR of 2-(p-tolyl)-2,3-dihydroquinazolin-4(1H)-one**

**2-phenyl-2,3-dihydroquinazolin-4(1H)-one:** ^1^H NMR (400 MHz, CDCl_3_): δ_H_= 8.27 (s, 1H), 7.62-7.60 (d, *J*= 8 Hz, 1H), 7.50-7.48 (d, *J*= 4 Hz, 2H), 7.40-7.34 (m, 3H), 7.25-7.22 (t, *J*= 8 Hz, 1H), 7.10 (s, 1H), 6.75-6.74 (d, *J*= 4 Hz, 1H), 6.68-6.65 (t, *J*= 8Hz, 1H), 5.75 (s, 1H) ppm (Figure S3).


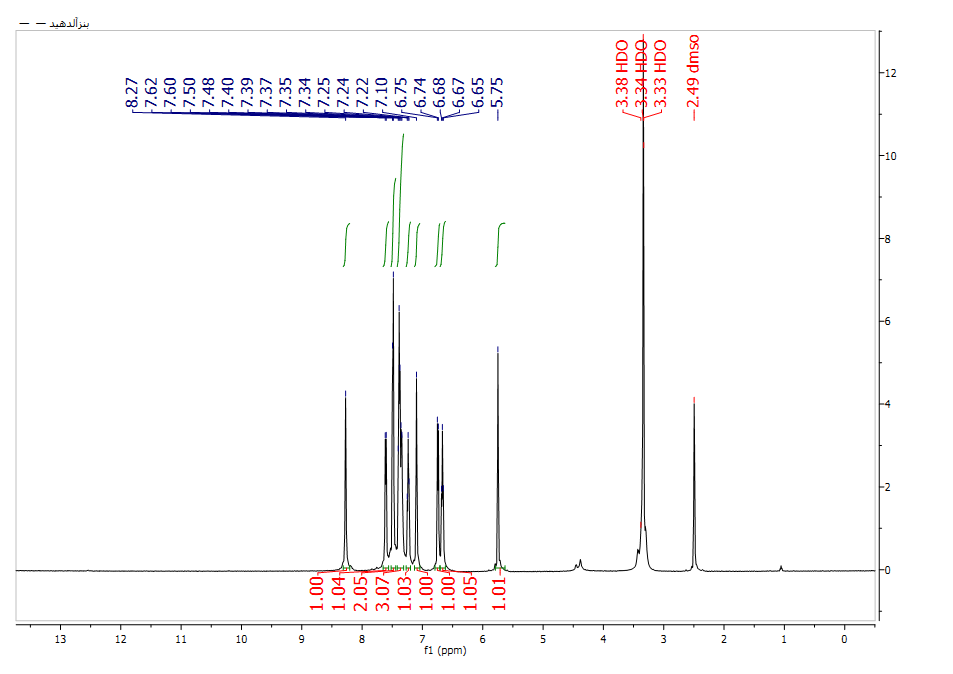


**Figure S3. ^1^H-NMR of 2-phenyl-2,3-dihydroquinazolin-4(1H)-one**

**ethyl 2,7,7-trimethyl-4-(3-nitrophenyl)-5-oxo-1,4,5,6,7,8-hexahydroquinoline-3-carboxylate:** ^1^H NMR (400 MHz, CDCl_3_): δ_H_= 9.24 (s, 1H), 7.97 (s, 2H), 7.61-7.51 (d, *J*= 40 Hz, 2H), 4.96 (s, 1H), 3.96 (s, 2H), 2.50-2.43 (m, 2H), 2.33 (s, 3H), 2.20-2.17 (d, *J*= 12Hz, 1H), 1.99-1.96 (d, *J*= 12Hz, 1H), 1.12-1.09 (t, *J*= 8Hz, 3H), 1.00 (s, 3H), 0.82 (s, 3H) ppm (Figure S4).


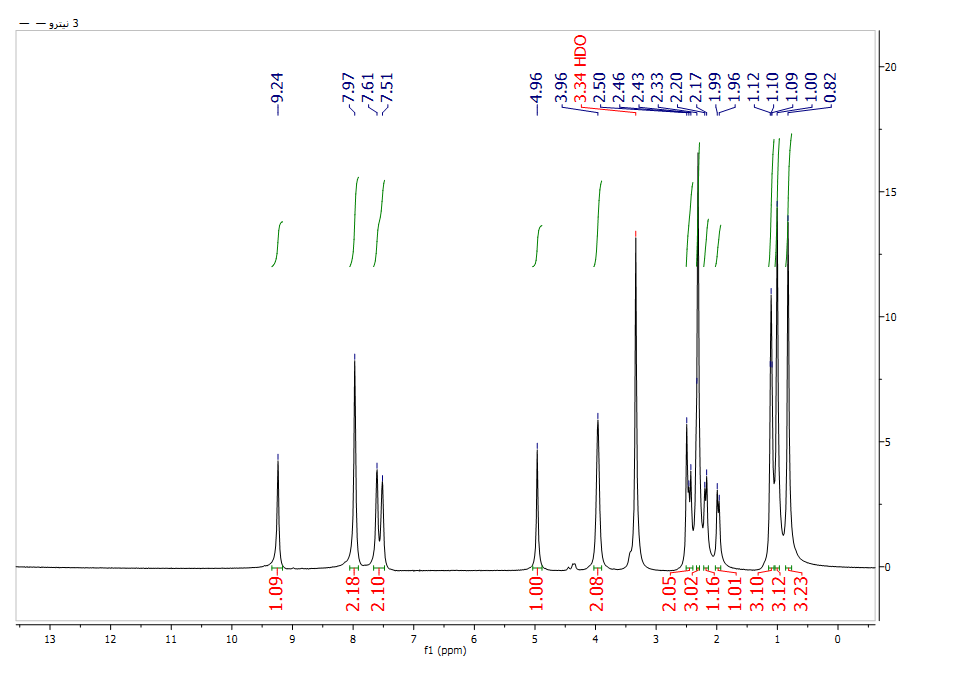


**Figure S4. ^1^H-NMR of ethyl 2,7,7-trimethyl-4-(3-nitrophenyl)-5-oxo-1,4,5,6,7,8-hexahydroquinoline-3-carboxylate**

**ethyl 4-(4-methoxyphenyl)-2,7,7-trimethyl-5-oxo-1,4,5,6,7,8-hexahydroquinoline-3-carboxylate:** ^1^H NMR (400 MHz, CDCl_3_): δ_H_= 9.00 (s, 1H), 7.05-7.03 (d, *J*= 8 Hz, 2H), 6.74-6.72 (d, *J*= 8 Hz, 2H), 4.78 (s, 1H), 3.97-3.95 (d, *J*= 8 Hz, 2H), 3.66 (s, 3H), 2.49 (s, 2H), 2.26 (s, 3H), 2.16-2.13 (d, *J*= 12Hz, 1H), 1.98-1.94 (d, *J*= 16Hz, 1H), 1.14-1.11 (t, *J*= 8Hz, 3H), 1.00 (s, 3H), 0.88 (s, 3H) ppm (Figure S5).


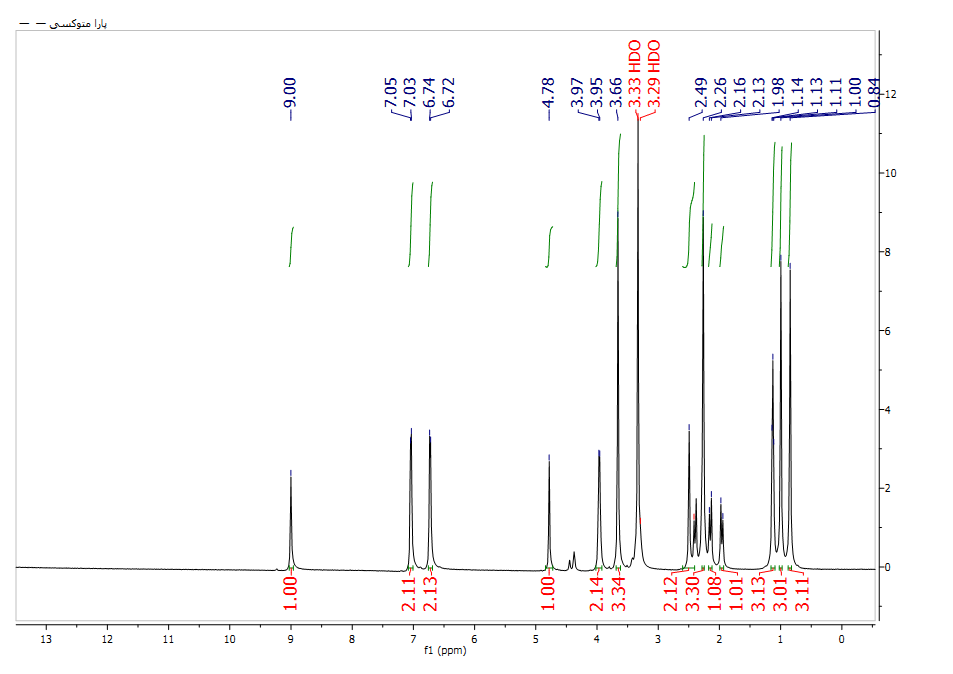


**Figure S5. ^1^H-NMR of ethyl 4-(4-methoxyphenyl)-2,7,7-trimethyl-5-oxo-1,4,5,6,7,8-hexahydroquinoline-3-carboxylate**

1. ^^ [↑](#footnote-ref-2)
